# Intravascular Large B-Cell Lymphoma Presenting as Systemic Capillary Leak Syndrome With Immunological Phenomena: A Case Report

**DOI:** 10.1155/crh/9919411

**Published:** 2025-09-11

**Authors:** Helena M. van Dongen, Bregje M. Koomen, Jordy Jurgens, Helen L. Leavis

**Affiliations:** ^1^Department of Rheumatology & Clinical Immunology, University Medical Center Utrecht, Utrecht, the Netherlands; ^2^Department of Pathology, University Medical Center Utrecht, Utrecht, the Netherlands; ^3^Department of Internal Medicine, University Medical Center Utrecht, Utrecht, the Netherlands

**Keywords:** autoimmune diseases, capillary leak syndrome, case report, intravascular large B-cell lymphoma, paraneoplastic syndromes

## Abstract

Intravascular large B-cell lymphoma (IVLBCL) has a high mortality rate, partly due to its heterogeneous presentation and rarity. We present a case of a 73-year-old woman who came into the emergency room in need of fluid resuscitation, interpreted as septic shock. However, broad-spectrum antibiotics gave no resolution, and no causative agent was found. Further physical examination showed proximal muscle weakness, Raynaud's phenomenon, and calcinosis cutis. During 3 weeks of admission, vasopressor support was required continuously due to a capillary leak syndrome. The patient passed away. The underlying malignancy was only revealed at autopsy. To the best of our knowledge, this is the first case of IVLBCL with hypovolemic shock due to systemic capillary leak syndrome in combination with a wide range of immunological phenomena.

**Summary**

• IVLBCL is a challenging diagnosis due to heterogeneous presentation and rarity of the disease.
• Upon exclusion of more obvious diagnoses, consider intravascular lymphoma in case of generalised oedema based on shock, elevated LDH and immunological phenomena.
• Early diagnosis and treatment might increase survival rate.
• Systemic capillary leak syndrome (SCLS) is a serious condition due to increased capillary permeability and is characterized by episodes of hypotension, oedema and hypoalbuminemia with hemoconcentration.
• SCLS can be primary (idiopathic) and secondary to for instance infection, malignancy, systemic diseases or vasculitis.

## 1. Introduction

Intravascular large B-cell lymphoma (IVLBCL) is a rare variant of non-Hodgkin lymphoma and is characterized by proliferation of malignant B-cells in the lumina of small to medium-sized vessels, leading to thrombotic and ischemic complications. The clinical presentation of this entity is heterogeneous due to the diversity of affected organs. This impedes timely diagnosis and treatment, contributing to its high mortality rate [1].

We present a case with an extraordinary combination of paraneoplastic or immunologic deviations. Tragically, only at autopsy, the underlying disease was found. Early recognition of IVLBCL is essential, as prompt treatment may improve outcomes. We aim to raise awareness among clinicians, since this rare condition can be encountered by healthcare professionals across various specialties.

## 2. Case Presentation

A 73-year-old female was brought to the emergency department with a 1-week history of progressive tiredness, muscle weakness, nausea, vomiting, and abdominal ache. She had no relevant medical history, but she mentioned muscle weakness progressive over the last year, and a biphasic Raynaud phenomenon of the hands for 3 years, progressive over the last few months. She had no night sweats, weight loss, or fever. On presentation, the patient had tachycardia and hypotension with normal to low central venous pressure, normothermia, and a stable respiratory state. She had pitting edema reaching above both knees and proximal muscle weakness in the lower extremities. There was no palpable lymphadenopathy. Examination of the hands showed calcinosis cutis, and no other skin abnormalities, especially no scleroderma, were observed. Initially, sepsis without a clear infection focus was suspected, and the patient was treated empirically with ceftriaxone and fluid resuscitation.

### 2.1. Investigations

Blood test results on admission (and after 3 weeks) are shown in Table 1. Main findings were thrombocytopenia, leukocytosis with neutrophilia, hemoconcentration, hyperlactatemia, hypoalbuminemia, elevated serum lactate dehydrogenase (LDH), creatine kinase (CK), and C-reactive protein (CRP). Urinalysis and urine sediment showed no significant abnormalities, particularly no significant proteinuria. Electrocardiogram showed a sinus tachycardia, and cardiac echography showed a normal pump function without significant valve dysfunction. Upon chest X-ray, there were no infiltrative abnormalities or cardiomegaly. CT neck, chest, and abdomen showed no signs of infection, malignancy, or lymphoma. Additional infectious workup consisted of blood, urine, and feces cultures, in addition to extensive serology, PCR, and QuantiFERON testing (for HIV, EBV, CMV, hepatitis A/B/C/E, parvovirus B19, lues, and tuberculosis). No active infection was detected.

**Figure 1:**
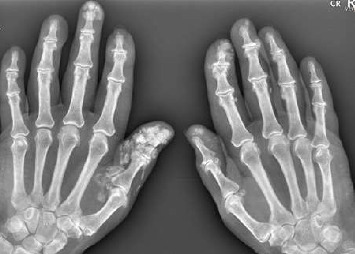
X-ray of the hands: multiple subcutaneous calcifications at the left digiti I and II and the right digitus II. To a lesser extent at right digiti I and III., this can be a part of, for example, dermatomyositis or systemic sclerosis.

**Figure 2:**
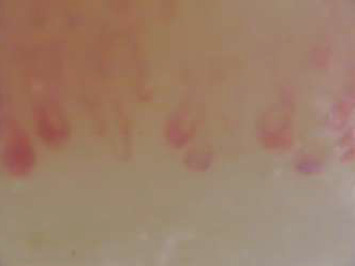
Nailfold capillaroscopy of left digitus IV; a representative image with decreased capillary density and mega capillary, classifiable as a scleroderma pattern.

Additional examinations were carried out, following diagnostic clues, which are discussed as follows. Hand X-ray showed calcinosis cutis (Figure 1), and nailfold capillaroscopy showed a scleroderma pattern (Figure 2). A PET-CT showed no signs of malignancy, lymphoma, or vasculitis. The CT chest showed no abnormalities in the lung parenchyma, and specifically no signs attributable to scleroderma such as interstitial lung disease, esophageal dilatation, or calcinosis cutis. Because of the muscle weakness, an MRI was carried out and showed a large quantity of edema in the musculus quadriceps femoris and back muscles, but no enhancement, indicating inflammation. Clonality analysis on peripheral blood was performed, but the results came in postmortem. The total IgG was not raised, but a monoclonal gammopathy (IgM Kappa 4 g/L) was found. The free kappa/lambda ratio was within normal range. Despite that, autoimmune serology showed a weak positive antinuclear antibody (ANA), and no specific detectable autoantibodies were detected.

### 2.2. Differential Diagnosis

The presentation of symptoms was compatible with distributive or third-space loss hypovolemic shock, supported by low to normal central venous pressure. Distributive shock, specifically septic shock, was evaluated and empirically treated, but without result. Blood cultures and extensive microbial investigation did not reveal a causative agent. Possible adrenal insufficiency was addressed with a hydrocortisone pump for several days, without improvement. The preexisting pitting edema was progressive upon therapy. Due to a lack of significant proteinuria on urinalysis, additional testing such as 24 h urine protein or urine protein electrophoresis was not carried out but could have raised suspicion of amyloidosis. Hemoconcentration and hypoalbuminemia, present at first presentation, were progressive. The combination of hypotension, progressive hemoconcentration, and hypoalbuminemia without proteinuria, with extensive edema, pointed to a systemic capillary leak syndrome (SCLS) [2]. The monoclonal gammopathy (IgM kappa, low titer) can also be found in this entity. Whether this contributes directly to pathogenesis is unclear [2].

SCLS should not be confused with systemic inflammatory response syndrome (for example, in the case of sepsis, acute pancreatitis, hemophagocytic lymphohistiocytosis [HLH], cytokine release syndrome, and more) [3]. In this case, HLH might be considered because of the thrombocytopenia, hypoalbuminemia, and capillary leakage, yet serum ferritin and sCD25 were not elevated, arguing against this diagnosis, and therefore, bone marrow biopsy was not performed [4]. SCLS can be idiopathic (Clarkson's disease) or triggered. A recent review states that 51% of SCLS is drug-induced, mainly due to antitumoral agents [3]. This patient did not use any of these high-risk drugs. 43% of SCLS is malignancy-associated, of which 61% is hematological (for example, non-Hodgkin lymphoma and multiple myeloma), and only rarely solid cancers. In this specific case, malignancy and especially lymphoma were highly considered, as explained in the following. A small subset of SCLS is infection-induced, mainly due to viruses, for example, dengue. SCLS associated with autoimmune/autoinflammatory disease is rare. Cases of psoriasis or its treatment are linked with SCLS; systemic lupus erythematosus (SLE) and antiphospholipid syndrome have been rarely associated with SCLS [3]. The patient had no signs of these classifying autoimmune diseases. Yet, we did record features of systemic sclerosis (SSc) and inflammatory myopathy. However, the acute presentation with SCLS, without scleroderma, with only a weak positive ANA, without specific antibodies, is not compatible with merely SSc. We found one case report with SSc and SCLS, but that patient had a coincidental nasopharyngeal carcinoma [5].

It has been postulated that whenever signs of SSc coincide with hematological disturbances such as monoclonal proteins, especially when the hematological symptoms develop shortly after signs of systemic sclerosis, the differential diagnosis should be focused on a paraneoplastic syndrome [6]. Therefore, in addition to the high LDH and lactate, in this case, a paraneoplastic syndrome, especially due to lymphoma, was foremostly considered. Apart from FDG PET-CT imaging not showing abnormalities, a random skin biopsy for the detection of intravascular lymphoma was considered but not carried out because of anticipated problematic wound healing due to the extensive edema. In hindsight, this should have been performed.

### 2.3. Treatment and Outcome

During the 3 weeks of admission, the patient was repeatedly admitted to the intensive care unit and empirically treated with antibiotics for presumed septic shock without clinical improvement. She received extensive fluid resuscitation with Ringer's lactate, albumin infusion, hydrocortisone infusions, and vasopressor treatment with noradrenaline. The patient had gained over 20 kg of bodyweight in the first week of admission, attributed entirely to the extensive edema. Despite extensive resuscitation, she developed multiorgan failure. Due to unbearable and irreversible suffering, she finally elected to discontinue further treatment. Within a few hours, she passed away.

Shortly after cessation of treatment, the peripheral blood immunophenotyping analysis revealed the presence of 0.2% CD45+ cells and 14/μl monoclonal CD19+ CD20+ CD21 weakly +, CD22+ IgM Kappa B-cells. These cells showed marker loss of CD5, CD23, CD10, CD103, IgD, and lambda. There were no signs of clonal T-cells. Concluding, a B-cell clone was reported.

**Figure 3:**
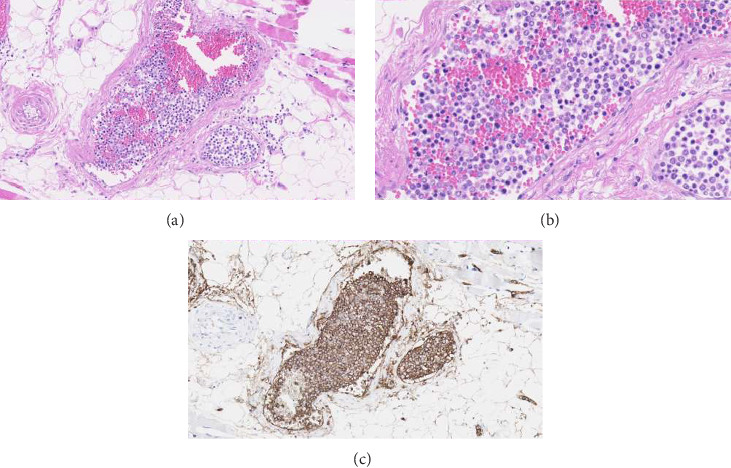
Atypical blast cells located in the lumina of small vessels of the musculus quadriceps femoris (left). (a) Hematoxylin–eosin (HE) staining (20x magnified). (b) Blast cells in more detail (HE staining, 63x magnified). (c) CD20 staining.

Autopsy was performed, and in multiple organs (heart, lung, liver, esophagus, stomach, colon, pancreas, kidneys, uterus, skin, skeletal muscle, leptomeninges, and dura), an intravascular proliferation of atypical blast cells with enlarged, polymorphic nuclei and a high nuclear/cytoplasmic ratio was found. These atypical cells showed strong immunohistochemical CD20 surface staining (Figure 3) and weakly positive BCL2 staining. The histopathological findings were consistent with an IVLBCL, with extensive involvement in multiple organs. No signs of amyloidosis were found.

We concluded that the systemic capillary leakage with hypovolemic shock and diverse immunological symptoms and the monoclonal gammopathy (IgM Kappa) were paraneoplastic, secondary to the lymphoma.

## 3. Discussion

IVLBCL is a rare type of lymphoma, with proliferation of malignant B-cells in small and medium-sized vessels. In more than two-thirds of cases, it is diagnosed upon autopsy [1]. Edema is a rare presenting symptom, seen in less than five percent of patients in a case series of 38 patients with IVLBCL [7]. To our knowledge, only three cases of IVLBCL presenting with SCLS with extensive edema and shock have been published before, unfortunately for all of these three cases, just like this case, with a postmortem diagnosis [8–10]. Immunological phenomena as presenting symptoms of IVLBCL have been reported five times before, including three cases with autoimmune hemolytic anemia [11–13], and two cases with small-vessel vasculitis [14, 15]. This case presents with calcinosis cutis, proximal muscle weakness, Raynaud, and nailfold abnormalities, which have not been reported previously in the context of IVLBCL.

SSc can be complicated by several types of cancers, including hematological malignancies. The most frequent neoplasm associated with SSc is lymphoma. To date, no characteristics of SSc can predict which patients are at risk for hematological malignancies [16]. For solid cancers, increased risk is seen in RNA-Pol-III positive patients [17]. Surveillance of SSc patients should be addressed [6, 18]. In this case, the patient did not meet the criteria for SSc diagnosis, and the immunological phenomena presented should be seen as a paraneoplastic syndrome.

Malignant cells in IVLBCL do not express molecules essential for extravasation or parenchymal invasion [1], yet high expression of molecules critical for adhesion and aggregation of these cells in the lumina of vessels is seen [19]. This can lead to the thrombotic and ischemic complications of IVLBCL, and possibly also to endothelial damage, and maybe linked to the secondary capillary leakage [7, 20], as seen in this case.

Diagnosing IVLBCL is challenging due to the heterogeneous clinical presentation and false-negative biopsies. Only in a minority of cases, the diagnosis is confirmed histopathologically (in bone marrow, skin, kidney, or other organs). Skin biopsy confirms diagnosis in only 10% of cases [21] and bone marrow biopsy in 32%–75%, which is also useful for staging [1]. Clonality analysis on peripheral blood demonstrated a B-cell clone in 5%–24%, in two case series of, respectively, 38 and 96 cases [7, 22].

In retrospect, earlier consideration of a paraneoplastic syndrome based on the combination of immunological features, shock, generalized edema, and elevated LDH might have prompted random skin biopsy, bone marrow investigation, and earlier clonality testing. However, earlier recognition most likely would not have changed the outcome for this patient, due to the very rapid course of disease and extensive organ involvement. Nonetheless, increased awareness could improve outcomes for future patients.

## Figures and Tables

**Table 1:** Laboratory results at admission and after 3 weeks.

Parameter	At admission	After 3 weeks	Reference range
Hemoglobin (mmol/L)	10.4	7.6	7.5–10.0
Hematocrit (L/L)	0.56	—	0.35–0.45
Platelets (×10^9^/L)	60	48	150–400
Leukocytes (×10^9^/L)	24.5	12.2	4.0–10.0
Neutrophils (×10^9^/L)	20.0	—	1.5–7.5
CRP (mg/L)	68	62	0–10
Lactate (mmol/L)	6.3	12.7	0–2.2
LDH (U/L)	1546	910	134–225
CK (U/L)	3929	103	< 35
Albumin (g/L)	21.6	16.2	35–55
ANA	Weak positive	—	Negative
Anti-dsDNA (IU/mL)	0.5	—	0.0–15.0
Line blot/myositis blot	Negative	—	Negative
IgG total (g/L)	5.72	—	7–16
M-Protein	—	IgM-κ 4 g/L	—
Free *κ* (mg/L)	—	34.57	3.30–19.40
Free *λ* (mg/L)	—	22.84	5.71–26.30
Free *κ*/*λ* ratio	—	1.51	0.26–1.56

*Note:* No other abnormalities were observed in leukocyte differentiation or peripheral blood smear.

Abbreviations: ANA = antinuclear antibody; CK = creatine kinase; CRP = C-reactive protein; Ig = immunoglobulin; LDH = lactate dehydrogenase.

## Data Availability

Clinical information relevant to this case report can be obtained from the corresponding author upon reasonable request. All data supporting the case are contained within the manuscript.
